# Exploring the generic skills required for the employability and professional wellbeing of Pakistani Millennials: The employers' perspective

**DOI:** 10.3389/fpsyg.2022.1070267

**Published:** 2023-01-05

**Authors:** Jawad Iqbal, Aliya Ahmad Shaikh, Warda Najeeb Jamal, Kalsoom Akhtar, Rabia Rahim, Shazia Kousar

**Affiliations:** Institute of Business, Management, and Administrative Sciences, The Islamia University of Bahawalpur, Bahawalpur, Pakistan

**Keywords:** generic employability skills, Pakistani Millennial graduates, professional wellbeing, thematic analysis, hard/technical skills, soft skills

## Abstract

**Introduction:**

This study aims to elucidate the employers' perspectives on the key generic employability skills which would enable them to seek graduate jobs and will ensure their professional wellbeing once they enter the job market after acquiring a University degree.

**Methods:**

In order to pursue this objective, an exploratory qualitative inquiry was deployed which involved two panel-based discussions. The respondents of each panel discussion were invited through the platforms of the Bahawalpur Chamber of Commerce and Industry (BCCI), and the Chamber of Commerce Rahim Yar Khan. These respondents represented Pharmaceuticals, Agrichemical manufacturers (fertilizers, pesticides), Livestock, Cotton, textiles, and apparel industry which are the main industries in Southern Punjab, Pakistan.

**Results and discussion:**

For the purpose of analysis, a thematic analysis was done in the context of grounded theory. Resultantly, the findings of the study reveal an employers' perspective on 10 key generic employability skills as must-have for a Pakistani Millennial graduate including various soft and hard skills, such as-Emotional Intelligence, Flexibility and Adaptability, Multitasking, Computer Literacy and Digital Skills, Information Literacy and Data Analytics, Oral and Written Communication in English and Urdu, Critical Thinking, Positive Politics, Work Ethics and Professionalism, and Commercial awareness. Hence, the study produces implications for the employability stakeholders, including government and academia for a much needed shift from a mere subject-based curriculum to a skill-oriented curriculum and training in the Universities, particularly in the region of Southern Punjab, and all across Pakistan as well.

## Introduction

In Pakistan, a lot of policy level discussions are directed at Youth Employability for the fact that the country is at the critical juncture of the “youth bulge” which contributes to a favorable position of demographic dividend in the country. About 64% of the population comprises the Youth (15–29 years), among which 41.6% represent the total labor force of the country (Nadeem, 2021). According to the report of the Planning Commission of Pakistan, the “youth bulge” in the country is predicted to rise by 2045 (Planning Commission Ministry of Planning Development and Reform, Government of Pakistan, 2017). Regarding it as a window of opportunity, numerous reports (like that of USAID) have emphasized an urgency for catering the mechanisms for human capital development, so that the country can reap the maximum benefits of this “demographic dividend.” Therefore, the strategic concerns for the country's policies lie around what will be the future of new job incumbents (Arif and Chaudhry, 2008), and what can be done to make them market-ready and ensure their professional wellbeing and sustainable employment.

Presently, what makes “Employability” a matter of concern lies in the fact that Pakistan's unemployment rate increased in 2020 to 4.65%. The recent United Nations Development Program Labor Force Surveys portray that the age bracket which remains most prone to unemployment includes the ones from 19 to 34 years (Nadeem, 2021). Which, according to Shaikh et al. (2021a,b) generational classification of the Pakistani workforce, comprises Pakistani Millennials/generation Y (born in 1982–2001; current age 21–40 years as of 2022). Normally, in Pakistan, it is ~21–22 years of age when a graduate completes their 16 years of education from the Universities. Thus, the UN figures of the age range for unemployed youth make it evident that a majority of qualified university graduates fail to seek employment in Pakistan after completing their university degrees.

As a matter of fact, most employers in Pakistan have accused universities of being unable to upskill and produce market-ready/job-ready/employable graduates and complain about skill deficits. This difference between employers' expectations and employees' abilities is viewed as a skill gap in the eyes of employers (Bano and Vasantha, 2019; Tan et al., 2022). Butt (2020) quoted a 2019's survey report to highlight that 78% of employers in Pakistan posit dissatisfaction with the skill set of fresh graduates.

The employers' concerns about skill deficit are reported on various forums for the fact that they compete in a dynamic external business environment where there is a constant flux in socio-economic conditions (Kuzminov et al., 2019), new political and strategic drivers (Blackmore, 2015), organizational shifts and enterprises (De Jager, 2004), diversification, globalization (Buntat et al., 2013; Mansour and Dean, 2016), fast-paced technological advancements (De Jager, 2004; Bano and Vasantha, 2019) such as automation (Chitra et al., 2021), business re-engineering (Herbert et al., 2020) and the pressures resulting from business re-modeling in post-COVID era (Mahajan et al., 2022). Certainly, they have induced an impact on labor market trends, work practices, and industrial employment patterns (Herbert et al., 2020; Mainga et al., 2022). Having faced the repercussions of these risky, complex, and turbulent circumstances (McCabe, 2010; Mainga et al., 2022), the employers of today have grave concerns about the new skill sets which will be beneficial for them (Bano and Vasantha, 2019), particularly in the era of “new normal.”

Nonetheless, the debate for upskilling Pakistani Millennial graduates is not as simple as it seems. Malik and Ameen (2021) have asserted that the professional landscape of Pakistan in both public and private sectors is evolving with the new nature of jobs. This has caused employers to view soft or generic skills as equally important as that other occupational skills (Buntat et al., 2013; Mansour and Dean, 2016; Sharma, 2018). Therefore, we have surpassed the era when the debate on the in-demand portfolio of skill sets was limited to hard or technical/occupational skills (Mansour and Dean, 2016).

A few generic skills are reported in the study conducted by Raza and Naqvi (2011) which portrayed the dissatisfaction of Pakistani employers (from sectors including banking; sugar; cement; food; auto; synthetics; leasing; IT; glass and ceramics; paper and board; oil and gas; and tobacco sectors of the economy) on the areas of personal development skills, intellectual development skills, social development skills, and professional development skills. These areas were inclusive of specific skills, such as intellectual development skills which refer to analytical ability, evaluation, knowledge development, diversity management, problem-solving, critical thinking, assessment, knowledge management, learning, and decision-making. Personal development skills (PERDS) include communication, teamwork, confidence, interpersonal affairs, information literacy, compare and contrast ability, workplace behavior, personality development, and information and communication technology. Professional development skills (PRDS) comprise forecasting, conflict management, customer service, fairness, leadership, job preparedness, professionalism, subject knowledge, and social development skills including ethics, socialization, and citizenship.

Therefore, nowadays, the acquisition of generic skills has turned out to be equally important as that of specific skills for the graduates' professional wellbeing in their careers (Selvadurai et al., 2012) and sustainable employment (McCabe, 2010).

Nonetheless, in Pakistan, the educational institutes responsible for fostering these skills have overlooked the industrial trends and requirements for skills (Kemal, 2005). Consequently, despite having university educational degrees, graduates face hurdles in seeking employment opportunities due to skill deficits. Certainly, it posits a question mark for if university-based education instills skills in graduates of today. If so, are the graduates trained on such market-centric skills which guarantee them a smooth market penetration, professional wellbeing, and sustainable employment. In order to address these concerns and facilitate universities, it study explores the local markets' skill requirements so as to dwell on local jobs.

For this purpose, Yorke and Knight (2006) have pointed out a dire need for “need analysis” which must be conducted by Higher Education providers in order to find out the skills that make students employable and how these can be embedded in the curricula, to achieve desired employability. This has also been emphasized by several researchers such as De Jager (2004), Jonck (2014), Mikalef et al. (2018), and Zhao and Kularatne (2020), who have asserted that without a clear understanding of the skill requirements of the industry, it would be nearly impossible to bridge the gap between the education delivered at universities and skills required by the market. In other words, bringing an industry-based perspective through proper need analysis will highlight the missing skillsets which might be overlooked by the supply side of employability i.e., Universities (De Jager, 2004; Jonck, 2014).

Already, the research employability literature is deficient in the context of Pakistan regarding the employers' perspectives on the skill sets (Sarfraz, 2020) which are needed to be inculcated in the Higher Education Curricula so as to align the work capacity of the labor force with the job market so that they may have a better opportunity of sustainable employment and professional wellbeing. Hence, the study was designed with the aim of market-based need analysis of employability skills by eliciting the employers' perspective on the generic skill set of graduates in field reality, so as to reflect on the industrial requirements of the various soft and hard skill elements of generic skills which would make a graduate employable and professionally equipped.

Owing to this gap, a project was fashioned to train the university graduates belonging to the Pakistani Millennial generation (born in 1982–2001) on the key generic skills (both hard and soft skills) which would ensure their employment and professional wellbeing in entry-level graduate jobs. As a part of this project, this study was designed as an initial market-based need analysis phase for determining the employers' needs for employability skills for entry-level graduate jobs. With reference to this, the research question for this study was formulated as:

RQ. What are the key generic employability skills which are required by employers for entry-level graduate jobs in the job market?

Concerning this question, this study obtained the perspective of the employers through a panel-based exploratory qualitative inquiry which focused upon the investigation of the employers' perspective on key generic skills that must be possessed by Pakistani Millennial graduates. Regarding this, the Chamber of Commerce of Bahawalpur and Rahim Yar Khan in Punjab, Pakistan were utilized as a platform to invite the employers/recruiters for this project-based study. The analysis of this study was based upon thematic analysis of the transcribed interviews and identification of key themes. The six themes emerging from this analysis led to the identification of 10 market-based generic skills (hard and soft skills) which are required in the local market, in the post COVID era of the “new normal.”

## Literature review

### Theoretical background of the study

The theoretical debate on employability is centered on the Human Capital Theory since it encompasses education and skills acquisition as an enabler for economic prosperity and the wellbeing of graduates. Thus, human capital is known for the quality of knowledge, skills, health, and finances of employees (Islam and Amin, 2021). Certainly, for an individual to be regarded as a quality “human capital,” it is important for one to constantly acquire, adapt, and enhance personal attributes and skills (Oliver, 2015). The inclination of individuals to acquire skills related to career prospects requires a long-term strategy, with a particular focus on professional wellbeing (Hojda et al., 2022). Van Horn et al. (2004) endorsed that an employee's professional stature and occupational wellbeing rest on their professional competence. Therefore, the focus of this study was directed at soliciting the skill sets which enable a graduate to be more competent and assure their sound professional wellbeing.

#### The role of soft and hard generic skills in employability

Employability is mostly conceptualized in two ways, (i) individual employability and (ii) institutional employability. However, most of the definitions conceptualize employability as more of an individual attribute. Hillage and Pollard (1998) comprehensively define it as “having the capability to gain initial employment, maintain employment and obtain new employment if required.” Herbert et al. (2020) stated that an employee's employability potential is delivered by the competence in delivering a task is accompanied by certain behavioral dispositions and skills.

Kuzminov et al. (2019) asserted that the skill sets of employable human capital comprise Generic and Specific Skills, both. They described that Specific skills are developed by seeking focused education and work experience in a specific domain over years. For example, the certifications which are opted for specific jobs, such as Microsoft Certified Professional (MCP), are an indicator of the occupational qualification of a person. Other researchers such as Harvey (2001) and Yorke and Knight (2006) have emphasized more on such skills, knowledge, abilities, and other competencies which are company-specific, sector-specific, and discipline-specific.

On the other hand, the generic skills are usually referred to in the literature with numerous titles such as transferable skills, generic capabilities, basic skills, essential skills, soft skills, work skills, enabling skills, key skills, and core competencies (Lauder, 2013; Messum et al., 2015; Caraivan, 2016), and hence are mostly attributed with graduate attributes. Barrie (2004) defined generic skills as “the skills, knowledge and abilities of university graduates, beyond disciplinary content knowledge, which are applicable to a range of contexts.”

Categorizing Generic Skills into three categories of General Human Capital, Kuzminov et al. (2019) described that General Human Capital 1 includes generic skills which are universal in nature, such as critical thinking, creativity, organization, and the ability to learn and work with others. Furthermore, they attributed non-cognitive traits such as Big-five personality traits, including Extraversion, Conscientiousness (awareness, responsibility, ability to follow and execute a plan), Agreeableness (ability to make consensus, friendliness), Emotional Stability (ability to make rational decisions during stressful situations), and Openness to Experience as part of the General Human Capital 2. Finally, they referred to the General Human Capital 3 with generic skills such as an individual's entrepreneurial ability which empowers one to form connections and collaborate with others for making the world a better place; also, it includes an individual's ability to transform institutions and social structures.

The researchers such as Yorke and Knight (2006) and Little and McMillan (2014) contend that although technical/disciplinary knowledge would provide an immediate entry into the workforce, generic skill sets pave a path for a lifelong career in the real world. For example, while technical skills such as knowledge and skills and qualifications related to social work, law, and engineering may determine an individual's work readiness; other generic skills and attributes such as organization, teamwork, and communication (Rowe and Zegwaard, 2017), networking (Bridgstock, 2017) and professional identity (Zegwaard et al., 2017) are conducive to finding requisite employment and sustaining it. Hence, a good combination of generic and specific skills is a requirement of the modern era (Mansour and Dean, 2016). Nonetheless, the focus of this study remained specifically on generic skills only.

It is worth mentioning here that generic skills further fall into two categories, soft skills, and hard skills. Zhao and Kularatne (2020) illustrated that Soft Skills are cognitive and interpersonal in nature which assures successful social integration in the workplace. Whereas, hard skills involve IQ and cognitive functioning in order to technically regulate various processes and tools. Therefore, Sharma (2018) regarded that as long as hard skills portray an individual's technical acumen, soft skills complement the technical skills by bringing in a people-oriented aspect. Hence, the exploration through this study involved both soft and hard skills which were generic in nature and were commercially in demand by employers.

## Materials and methods

### Research design

In order to delve into the generic skill sets (hard and soft skills) that may make a Pakistani Millennial graduate more employable and assure them professional wellbeing in the job market of the post-COVID era, an exploratory qualitative inquiry opted for this study. In order for it to be executed systematically, a grounded theory research design was undertaken. Although the grounded theory has been advocated for theory integration and development (Creswell, 2007; Chapman et al., 2015), however, the purpose of deploying this method was limited to yielding an industrial profile of skills. As, Strauss and Corbin (1998) advocate that grounded theory can also be used for systematic exploration, description, or conceptual ordering, rather than for a purpose of theory building only.

### Population

The study's population consisted of the employers/owners/recruiters from medium to large scale private enterprises/businesses, from two different cities of Southern Punjab, Bahawalpur and Rahim Yar Khan, belonging to Pharmaceuticals, Agrichemical manufacturers (fertilizers, pesticides), Livestock, and Cotton, textiles, and apparel industry.

### Sampling technique and sample size

The two panel-based discussions were joined by 26 respondents whose responses sufficed the point of saturation, which is endorsed by Creswell (2007), who asserted that for research based on grounded theory design, it is usually between 20 and 30 interviewees that the data starts saturating, the repetition starts, and no new themes emerge from the data. Out of these 26 respondents, a majority of 14 were from Agrichemical manufacturers; the Livestock industry was represented by three respondents, and nine respondents joined from the Cotton, textiles, and apparel industry.

These industrialists/employers/recruiters were contacted *via* the Chamber of Commerce of their respective cities. For example, the researchers collaborated with the secretaries of the presidents of the two Chambers of Commerce, and they facilitated providing the contact details of the business owners who had registered their businesses with them. This way, the business owners who provided their consent for availability and time were invited to the panel discussions. Hence, a convenience sampling technique was deployed for this study.

### Data collection

For the exploration of the employability skill sets, although the inquiry was initiated with the research question and objective of this study, following Chowdhury and Miah (2016) and Chowdhury and Miah (2019) approach to identifying employability skills, the employability skills were first sorted through the literature. Later, with the discussion of Recruitment and Selection Experts (who were mostly owners or businessmen of the enterprises' understudy), these skill sets were initially screened to a list of 36 important employability skills. All of these skill sets were incorporated in the interview protocol which was based on open-ended questions that mainly addressed the employers' preferences and expectations regarding the commercially relevant skills which are fundamental to the employability of Pakistani graduates' in entry level graduate jobs (Appendix 1). It is to be noted that the subject of discussion was limited to the employability skills of the “Pakistani Millennials/Generation Y” (born in 1982–2001) (as categorized by Shaikh et al., 2021a,b).

## Findings and discussion

**Figure 1:**
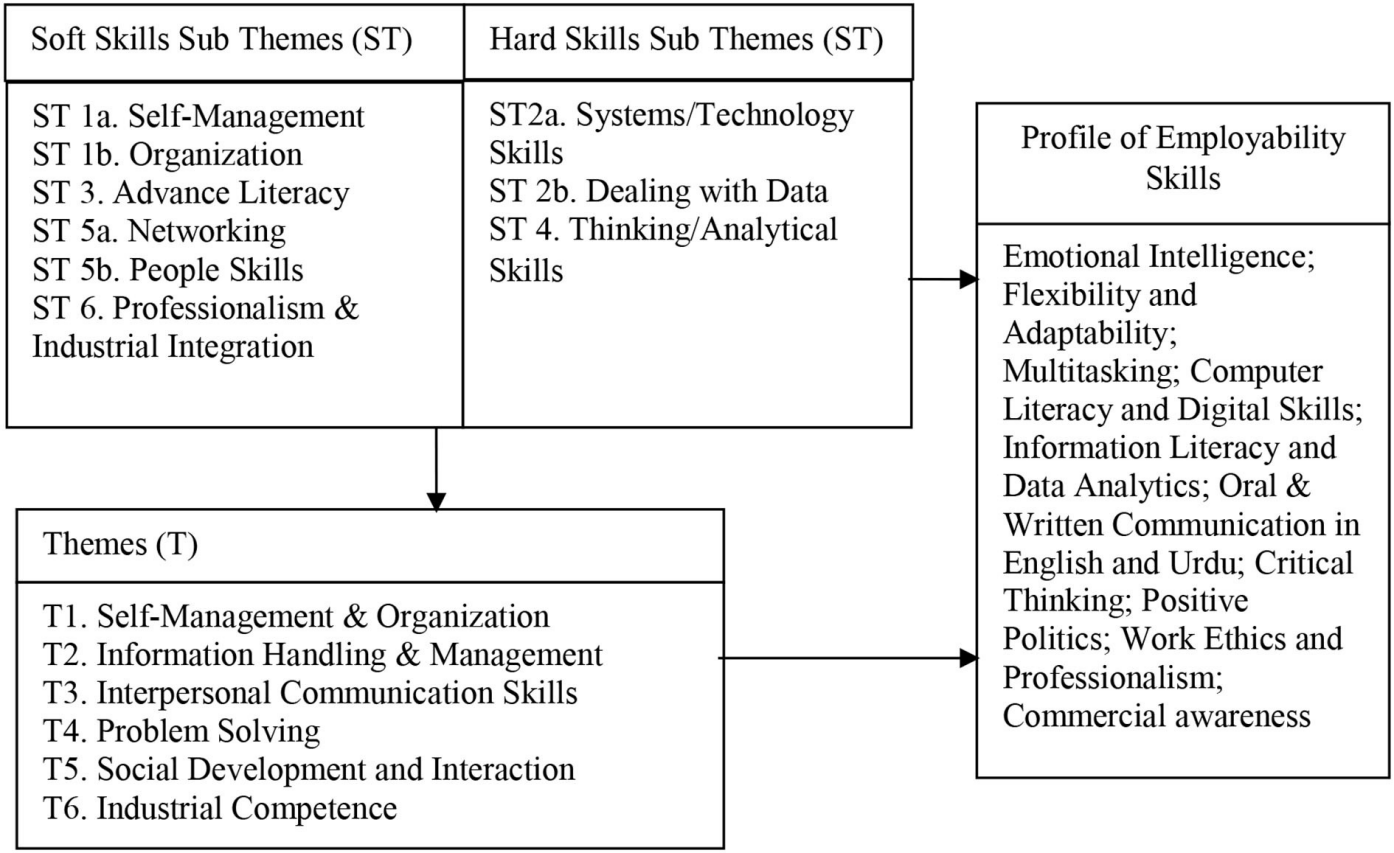
Conceptual framework exhibiting the relationship between generic skills' themes, sub-themes, and profile of hard and soft employability skills.

The qualitative data was analyzed through thematic analysis which followed the steps suggested by Braun and Clarke (2006) and Maguire and Delahunt (2017). Accordingly, the data items were first identified from the verbatim transcripts which were produced on the pattern of audio-recordings of the panel discussions. Later, in order to formulate codes, the transcripts were reread several times to locate distinct patterns in the data. Accordingly, a set of codes similarly depicting a pattern were placed in one sub-theme. This also involved the compilation and segregation of sub-themes according to the nature of the soft or hard skill. Subsequently, the sub-themes depicting soft and hard skill elements relevant to one theme were collated under the titles of distinct themes. Later, each theme was analyzed and defined. Finally, this led to the generation of the following six themes (1) self-management and organization, (2) information handling and management, (3) interpersonal communication skills, (4) problem solving, (5) social development and interaction, and (6) industrial competence. Altogether, these themes discussed 10 employability skills, which are required by Pakistani employers, as mentioned in Figure 1 of the study.

**Figure 2:**
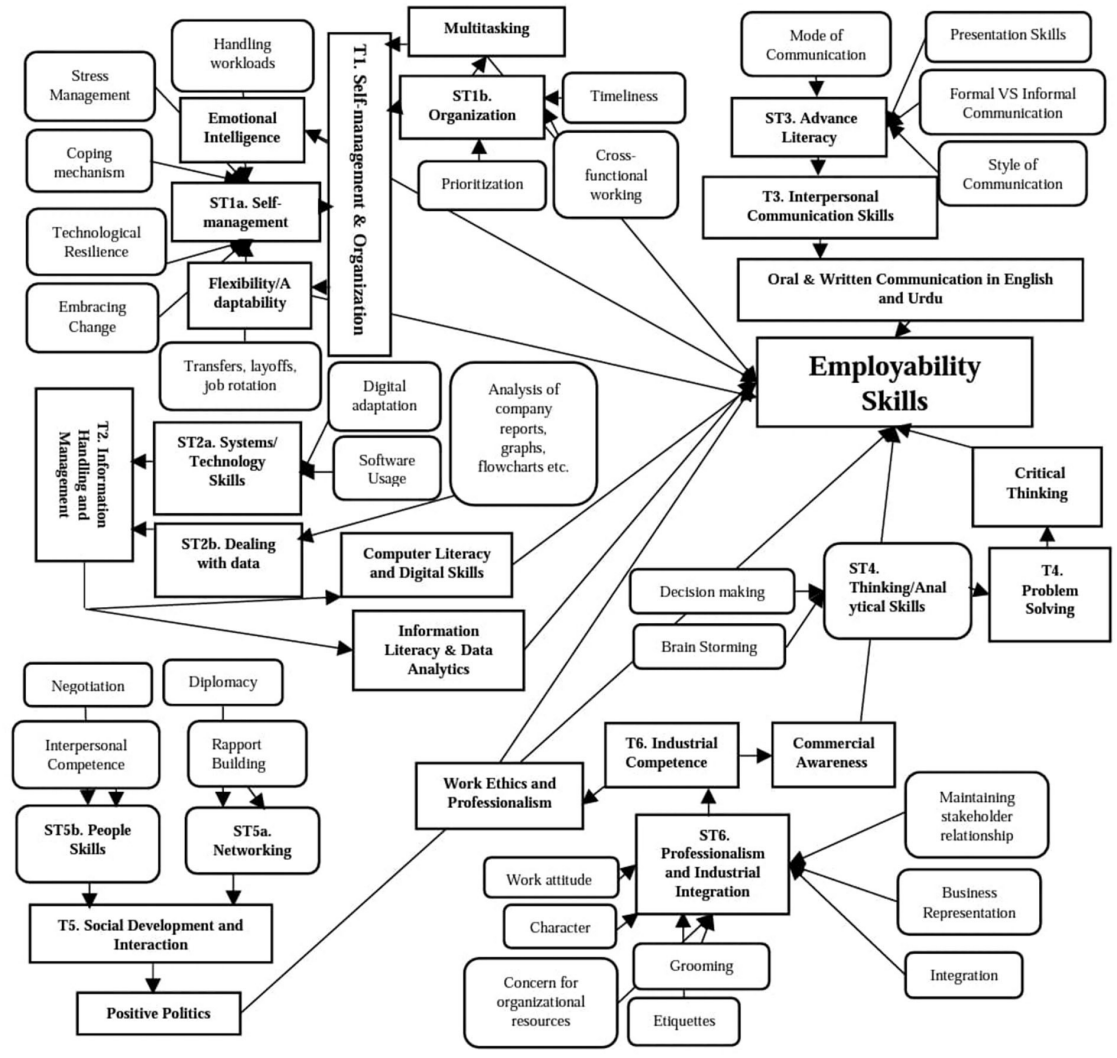
Thematic map illustrating the key codes under each sub-theme which are then compiled under distinct themes, thus each theme then directs to its associated employability skills.

Figure 2 portrays a thematic map which depicts how codes were assimilated into sub-themes, which then led to distinct themes depicting their associated employability skills.

Below is the analysis of each theme under separate heads:

### Theme 1. Self-management and organization

The first theme emerging from the thematic analysis depicted the employers' perspectives on self-management and organization skills which must be displayed by the graduates who have recently undertaken a university degree. Although, the employability studies have mentioned several other skill elements as fundamental to self-management abilities, such as punctuality, grooming, empathy, commitment, dependability, abiding by the rules, planning and organizing, emotional intelligence, and the ability to take initiative (Fraser et al., 2019), cooperation with others, self-reliance, overcoming shyness, ability to take learning initiatives, experimenting, independently executing tasks, staying updated about general knowledge (Selvadurai et al., 2012). However, the emphasis of the panel's respondents was restricted to the discussion over soft skill elements of Emotional intelligence and Multitasking. Furthermore, the respondents stressed flexibility/adaptability as a key soft skill essential for people to self-organize and embrace change.

#### Emotional intelligence

The first skill highlighted in the panel discussion turned out to be “Emotional Intelligence” (EI). The literature documents Emotional Intelligence encompasses several socio-emotional competencies such as self-awareness, regulation of emotions, motivation, empathy, and social skills which are essential for the workplace wellbeing of an employee (Aloui and Shams Eldin, 2020; Tolbert, 2020). Almost all of these competencies were highlighted in various comments of the respondents, which portrayed that a majority of the employers favored the candidature of graduates for employability who possess a high degree of emotional intelligence competencies. As one respondent opined:

“*…I strongly believe that a candidate with a strong EI quotient comes up with better guts of situational awareness, self-awareness, and regulation of emotions. All of these traits contribute to better interpersonal functioning in groups and project teams…”* (Employer/Recruiter K)

About three-fourth of the respondents prioritized Emotional Intelligence as an essential skill complementing the ability to manage stress. Portraying the perspective on how Emotional quotient depicts the emotional wellbeing of an employee in stressful scenarios, work overloads, and conflicts, an interviewee opined:

“*…The stress triggers vary from person to person, it can be overwhelming work, negative politics in the organization, peer pressure or any other thing. As such, all of these situations require a sound internal appraisal to develop a coping mechanism which certainly requires a sound Emotional Quotient…”* (Employer/Recruiter B)

The discussion further revealed how interviewers expected that university education and socialization should build a sound Emotional quotient of a fresh candidate. This was highlighted by a respondent who quoted from his experience as a selection interviewer:

“*…While we expect a university education, socialization, and group work experiences should build a sound Social Intelligence and Emotional Quotient in a graduate. Yet, most of the candidates mishandle situational which are emotionally charged. And if these candidates have ever been selected, they are found weak in dealing with teams, colleagues, senior management, managers, external clients, etc., and take time to learn and emotionally adjust with them…”* (Employer/Recruiter A)

Altogether, the responses portrayed that emotional intelligence is viewed as the employees' ability to be emotionally self-aware and emotionally resilient in stressful situations of conflict and work overload so as to perform with better People and Relationship Management skills.

#### Flexibility and adaptability

The next skill nominated by panelists was “Flexibility and Adaptability” which was viewed as a much-needed skill for framing long-term and sustainable careers for entry level graduates.

When asked about the experience of working with fresh graduates, the respondents opined their concerns about the lack of flexibility and resilience in the Pakistani Millennial generation. They demonstrated a majority of arguments regarding how a graduate, being a novice, fails to realize the importance of resilience and steadfastness in their early careers. Portraying this concern, an employer commented:

“*…I was unaware of how stringently employees lose morale in economic downturns and work pressure till I had to lay off the individuals exhibiting no resilience attitude at all…”* (Employer/Recruiter H)

For an explanation of what kind of flexibility and resilience was required by the industry, a recruiter opined:

“*…It must be understood as a coping mechanism which has a lot to do with your mental strength. It requires one to understand the things in a broader perspective, reorganize oneself and reframe things for good…”* (Employer/Recruiter J)

In the wake of the modern employment era, when most graduates face the hurdle of person-job fit and person-organization fit, another group of panelists collectively opined:

“*…In most situations we observe employees have to opt for jobs that have no direct relevance to their interest or degree domains, so in such cases also one can only learn and grow with flexibility…”* (Employer/Recruiter I)

Another majority of the respondents regarded flexibility as a must-have trait for one's adjustment to organizations that have their own established cultures. For that too is regarded to vary from organization to organization (Nair and Mukherjee, 2015). One response in this regard was observed:

“*…Practically, every organization has its protocols, values, attitudes, and beliefs, including the way they celebrate different events, practice collegiality, and perform in communities of practice. This culture develops with the passage of time. So, having flexibility will be quite useful for the prospective graduates to adapt to new environments and do networking with new people with diverse set of values, norms, and beliefs …”* (Employer/Recruiter H)

In general, the respondents of this study viewed it from the perspective of the cultural competence of an individual. With reference to this, one respondent considered that a graduate needs sound social intelligence for successful integration into the workplace's communities of practice. This was notified by an employer as:

“*…I think employees have to re-establish themselves after they enter a workplace. There is an organizational culture one is informed about on orientation day, and then one gets to experience it through exposure and social commitments. Certainly, it takes time as there is a constant evolution in the form of learning and unlearning …”* (Employer/Recruiter No. D)

In addition, employers voice their belief that the University's Extracurricular activities provide a medium for the development of flexibility and adaptability in graduates. Linking it explicitly to extracurricular activities, a respondent stated:

“*…Flexibility is not taught, it is a learned trait…In educational setups, Extracurricular activities are the best way to polish this trait. Once learned, it facilitates the job incumbent to adapt with unexpected changes of circumstances like transfers, relocation, change of job roles and work teams, etc. …”* (Employer/Recruiter F)

Thus, the key abilities associated with flexibility were found to be closely in line with Fraser's et al. (2019) study, who defines flexibility as an individual's ability to flexibility adjust in varying circumstances while keeping up a spirit of resilience.

#### Multitasking

The next skill highlighted by the panelists was “the ability to multitask.” Although, the ability to multitask has been quoted in the literature with two different points of view, where one view states it as a sign of efficiency in the contemporary era, while the other point of view regards it as a sign of distraction, disintegration, or ineffectiveness (Lin et al., 2012). Nonetheless, the discussion portrayed that a majority of employers in the panels contended that the modern world of work requires subject-matter specialist cum generalist roles. Most of them attributed it to the ability to be an “all-rounder performer” and considered it an essential requirement for most of the work-based scenarios of modern businesses. As one respondent elaborated:

“*…We do not underestimate the value of specialization, but we also cannot afford to overlook the modern job roles which require a knowledgeable individual with a broad array of expertise. This is why many organizations, nowadays, have a ‘Management Trainee Program' that involves a yearlong inter-departmental rotation before designating a department to the employee. This polishes the general skill sets of an individual, as such, these individuals simultaneously stretch on various tasks and roles …”* (Employer/Recruiter E)

This portrayed an essential aspect for the freshers who may regard themselves as proficient in a particular skill or discipline, yet an employer may feel that they are not capable to demonstrate the desired level of performance due to their inability to perform as allrounders. Nonetheless, those who the panelists believed were allrounders, also complained about the freshers' lack of ability to prioritize things, schedule, and manage them well in time.

“*…The work impression of a fresh job incumbent is also gauged by effective time management skills and control over tasks. Which I believe is quite possible to be done in modern technological era, where one can utilize various tools and gadgets to organize themselves by setting reminders, making online Gant charts etc. Often, we witness that Millennials would fail to make a tradeoff in various tasks according to the nature of resources required, urgency, importance, or they would lose focus on other things while working for one particular task…”* (Employer/Recruiter H)

Altogether, the respondents emphasized multitasking as an ability to be an allrounder who prioritizes, schedules, and executes tasks according to the need of urgency, importance, and resources; handles pressure in times of crisis; controls multiple tasks simultaneously, and uses tools and technology to manage time.

### Theme 2. Information handling and management

The second theme arising from the panel discussions turned out to be “Information handling and management.” Under this theme, a couple of hard skill elements including computer literacy, digital skills, information literacy, and data analytics were portrayed as key skill sets.

#### Computer literacy and digital skills

The findings further portrayed the importance of Information Literacy and Data Analytics as advanced skill sets which are viewed as valuable in the eyes of employers to meet the requirements of the organizational data. Altogether, the employers' perspective was aligned with the assertion of Bejaković and Mrnjavac (2020) who posited that digitization has caused polarization in the job market, where all the opportunities are for employees with digital proficiencies rather than the ones who are low-medium skilled and possess manual work proficiencies. This is particularly true in the case of Pakistan which earlier used to export cheap low skilled labor, but now is transitioning from the status of a middle-income country to a sustained production-based economy (Martinez-Fernandez and Choi, 2013). Hence, the new set of job roles requires more computer and digital proficiency.

When asked for it, a majority of the employers were focused on their viewpoint that they required a job incumbent with more than basic computer usage. They opined that graduates lacked hands on experience with software that they deployed in the industry. Similarly, one response was portrayed as:

“*…In Post-Covid era, we live in phygital workspaces where we largely incorporate digital solutions at our workplace, be it be HR softwares like Harmony, SAP, Orange HR, or any Customer Relationship Management Software or another. So, we would prefer for one to be acquainted with the usage…”* (Employer/Recruiter A)

Another employer put it as follows:

“*…We are living in a digital E-world. I do not see survival in the traditional job mindset because technology, the internet, and related fast-paced changes have revolutionized the work patterns and job dynamics. Here…we need workers possessing digital competency with advanced levels of digital skills. For example. Nowadays it has become mandatory for everyone to learn virtual collaboration skills so that they could manage meetings and collaborations via Zoom, Microsoft teams or any other software…”* (Employer/Recruiter I)

In addition, some of the respondents of this study did make some stipulations regarding the ever-evolving nature of technological revolutions which posits the requirement for updating computer and digital skills, from time to time. This has already been made evident by Pirzada and Khan (2013) that in the modern e-world, the professional employment of fresh Pakistani graduates depends upon how quickly they equip themselves with new ICT devices, and generic software tools, and keep up with the pace of upgradation in applications. Mainga et al. (2022) attributed it to one's propensity for technological resilience which would require one to keep updated with advanced technological trends.

#### Information literacy and data analytics

It was deduced from the discussions that in the modern era of a knowledge-driven economy and information revolution, it is nearly impossible to exist without having the skill to deal with technological gadgets. Malik and Ameen (2021) also asserted that in Pakistan, the future professional landscape will require a lot of information workers. The research literature also quotes this skill set as essential to cope with the requirements of megatrends like the fourth industrial revolution. This would involve employers seeking furnished skill sets to deal with Mechanization, Artificial Intelligence, Big Data, Automation, Data Analytics, Data Integration, Quantum Computing, etc. (Chitra et al., 2021).

The significance of “Information literacy and Data Analytics Skill” was stated in the panel discussion as follows:

“*…We live in an information-based, data-oriented, and knowledge-driven economy. Here, we need data scientists who use relevant scientific, technological, and mathematical knowledge to read and interpret manuals, portray information through visuals like graphs, and have the skills to transform data into actionable insight. Such individuals have the most expensive and in-demand skillset in the contemporary era…”* (Employer/Recruiter D)

A similar comment in this regard appeared on probing:

“*…Analytics acts is a quick tool to analyze facts and processes and generate solutions, make decisions and predict future. This involves a lot of designing and maintenance of data systems and databases…”* (Employer/Recruiter J)

Overall, it was demarcated as the ability to acquire data from primary and secondary sources, utilize statistical techniques for analyzing patterns and trends in data, provide reports with meaningful insights, and develop and maintain databases. However, it is worth mentioning here that only two fourth of the employers endorsed this skill as an essential competency for the starting phase of individuals' careers.

### Theme 3. Interpersonal communication skills

The third key theme addressed was interpersonal communication skills, which Williams et al. (2019) considered as a composite feature of rapport building, collaboration, and honesty, as essential for interaction with authorities, subordinates, peers, customers, and other stakeholders. However, the panelists limited their discussion to soft skill elements of Oral and Written Communication in English and Urdu. Several other researchers like Sharma (2018) also considered it an indispensable element in contemporary formal and informal work environments. The panelists of this study discussed it as follows:

#### Oral and written communication in english and urdu

Although language and eloquent communication are recognized as the first modality to qualify for a job (Caraivan, 2016), it was discovered through the results that employers struggled with seeking graduates with proficiency in the communication domain.

Overall the employers remained in the argument about the dual-language status of the country's official language. Although the English language gets much recognition in the country for being a symbol of status, quality of education, modernization, and socio-economic and cultural predominance (Shamim, 2011; Umrani and Bughio, 2017; Zaidi and Zaki, 2017); nonetheless, the respondents viewed the English language as the main hindrance in the communication skills of the graduates. A response made in this regard is stated as follows:

“*…Although we recognize both Urdu and English as official languages, nonetheless, unfortunately, our graduates cannot converse smartly in any of the two languages. Often, we find that the graduates fail to make good first impressions in various screening interviews, group discussions, presentations, etc. just because of English language constraints. While, for Urdu language, I have observed that the struggle is limited to written official correspondence, mainly…”* (Employer/Recruiter C)

However, the literature does not regard Urdu Language proficiency as a major issue because for official correspondence English dominates several domains such as legal services and proceedings of courts (Ahmad et al., 2020), science and technology, information, and across several other industrial and business sectors. Even that, the provincial and federal civil service examinations, and civil and military bureaucracy are carried out in English (Haque, 1982 as cited in Umrani and Bughio, 2017).

In addition, a few responses highlighted that the graduates remained ignorant of the intricate requirements of formal and informal communication. Such as they highlighted that graduates were not accustomed to the communication styles required in formal and informal situations and various cultures. In another research literature study, Nair and Mukherjee (2015) also realized that many business communication and meeting agendas fail if employees are not accustomed to the cultural norms and cultural variances across the organizations (Nair and Mukherjee, 2015).

All of these aspects were highlighted in several comments, one of which is stated as follows:

“*…It is very likely for a graduate to get into difficult conversations in convincing stakeholders (boss, client, peers), feedback meetings, and especially while communicating bad news. Most of the freshers do not realize the value of message tone, the significance of communication hierarchy which must be followed and the language of the message which should be tailored according to the stakeholder addressed in the message…”* (Employer/Recruiter B)

Similarly, concerning written communication, the employers quoted numerous places in which loopholes in written communication were observed with regard to grip over language, vocabulary, grammar, and sentence structures while formulating business proposals, report writing, and email writing. In this regard, an employer voiced his dissatisfaction regarding email communication through one comment, which is stated as follows:

“*…I have witnessed numerous incidents where the professional image of the sender is discredited in email communication when one forgets to use salutations or may commit other errors like the too formal or too informal tone of the message which distorts the purpose and impression; other than this typo, use of emojis or abbreviations and other such mistakes have made us lose our clientele several times…”* (Employer/Recruiter K)

Whereas, a majority of the comments with regard to oral communication were noted pertaining to presentation skills. Selvadurai et al. (2012) regarded that employers seek individuals with effective presentation skills which include the ability to deliver complex concepts in a simplified manner as per the requirements, needs, and interests of the concerned parties. Hence, an effective presenter utilizes a good combination of verbal and non-verbal cues to tailor content and make it understandable to the audience. Elaborating on a question regarding presentation skills, for if graduates could demonstrate it on degree completion, a respondent explicitly stated:

“*…At times we need to present information and company reports to the board. Here, we need employees with strong abilities to present information in a simplified manner, with visually appealing content. Then we need their persuasion skills in order to obtain positive responses from the stakeholders. Sometimes we need to convince them on our progress that we are striving for perfection; other times we need to convince them to modify standards, rules, and procedures. This also requires the character and credibility of the presenter. Whatever the case is, we need good presenters…”* (Employer/Recruiter A)

This response regarding proficiency in communication as the ability to persuade, exhibit empathy, and the ability to illustrate complex concepts in the form of simple and understandable has also been illustrated in the study of Ahmad Tajuddin et al. (2022).

Another respondent attributed communication skills to the ability to communicate technical knowledge and make it understandable to the stakeholders. It was comprehensively reported in a comment as follows:

“*…In a Service Industry, unlike that of mechanical procedures of manufacturing industries, socially mature individuals are required who are apt at communication and presentation skills. We need employees with a sound technical acumen, who present our services by understanding/reading the customer, exhibit empathy, tailor tones of the messages and generate a deal with the help of their persuasion power…”* (Employer/Recruiter D)

The findings are closely aligned with the assertion of Sharma (2018) who contended that besides being able to effectively communicate, there are numerous other complementing factors essential for closing commercial/business transitions including attributes like good customer rapport, business, and social etiquette, conversational skills, adaptability, credibility, and reputation.

When asked about the reason for the aggravation of language issues in graduates, a respondent highlighted:

“*…Language as a skill has been completely overlooked by Pakistani graduates. The technological advances and communication over mobile phone texts and emails involves a lot of usage of Roman English and short hand which has caused a deterioration in basic language …”* (Employer/Recruiter J)

Altogether, the discussions were quoted with several instances where the poor oral communication of graduates was observed by them in the workplace, including, occasions when a new job incumbent makes phone calls, delivers presentations, convinces stakeholders, writes emails, conducts face-to-face and online meetings, conference calls, negotiates with clients, and impromptu speaking situations in seminars. Whereas, with regard to written communication, the reservations were noted in the broader areas such as Resume Writing, Note Keeping, Email Etiquette, Memorandums, Instructions writing, Report Writing, Press release Writing, and Minutes Writing. On discussing this issue, an employer voiced his concern:

“*…We expect that a CV should speak for the graduate. S/he must be apt at writing minutes of the meetings and all the basics of email communication must be known prior to joining a workplace, and that too in English and Urdu (whatever correspondence language is used in the organization) …”* (Employer/Recruiter H)

In summary, proficiency in oral and written communication in Urdu and English was illustrated as the ability to professionally communicate by using appropriate language, communication style (formal vs. informal), and structuring message tone according to communication mode (verbal, written, e-mail, online, face-to-face meetings) and stakeholder addressed (clients/peers/superiors). Nonetheless, it was observed that although, literature portrays “listening” as a fundamental attribute of effective communication in addition to oral and written communication skills (as mentioned in Rasul et al., 2013), however, the findings of this study did not explore any such aspect.

### Theme 4. Problem solving

According to Fraser et al. (2019), problem-solving is defined as the ability to make an intelligent choice among alternative solutions to a problem, monitor the consequences of each alternative, sourcing resources, and helping to resolve a problem. Therefore, being regarded as a systematic process (Norshima, 2011), the respondents of this study considered good critical thinking as an essential prerequisite of problem-solving.

#### Critical thinking

Researchers such as Pearl et al. (2019) and Pardo-Garcia and Barac (2020) have stated that critical thinking is viewed as a higher-order thinking ability that allows one to self-reflect and systematically solve problems by critically analyzing the components of an issue and choosing an optimal alternative. Overall, the employers from the panels have regarded critical thinking as a highly sought-after quality among graduates for problem-solving. Regarding how the respondents of this study stated that they evaluated the reasoning ability of a prospective employee through interview questions, a comment was noted as follows:

“*…In order to evaluate a prospective employee on this highly sought-after employability marker, we often as interview questions like ‘Give me an example of a project that undertook in your student life. And then we talk through bits like ‘What happened when it went wrong?…”* (Employer/Recruiter F)

Portraying the dissatisfaction with the critical thinking skills of freshers, an employer accused the education system of failing to inculcate thinking skills in students and stated:

“*…The graduates lack the Value judgment call or critical thinking ability. The concept of critical thinking lies in the fact that anything can be true or untrue based upon the observer's experience, it would depend upon the reference point which is the observer's experience for if he/she believes in it or not…”* (Employer/Recruiter I)

A majority of the responses portrayed that employers did not find a correlation between formal education and the development of critical thinking skills. The respondents' view was conclusively put up by one interviewee as follows:

“*…Our intellectual criteria are dictated by the reasoning ability and reflective thinking of employees. We need people who are apt at analyzing problems from multiple dimensions, evaluating alternatives, weighing risks, interpreting and concluding solutions and future directions. Nonetheless, university education has failed to promote thinking skills in students. Even the students who have had numerous case study discussions, fail to critically evaluate the things and reproduce new solutions…”* (Employer/Recruiter G)

Portraying the reason for the lack of critical thinking ability in Pakistani graduates, a respondent stated:

“*…In Pakistan, most of the students obtain their degrees and pass their exams in the information duplication mode, and to internalize the information and critically reflect on it. It is similar to the situation like we would often listen people talk about constitution but no one actually knows about it. So, students remain in a duplicity mode even with our subjects. All they care is that we have to pass the subjects without critically analyzing the facts taught…”* (Employer/Recruiter G)

Further exploration revealed the loopholes in the university education which was considered a vital reason why graduates fail to exhibit critical thinking ability, as an employer stated in this regard:

“*…In our company, we have the KPI based performance evaluation. For each task, i.e. quality surveillance, hygiene documentation, cost related to sub-suppliers, and rebates etc., in order to achieve these KPIs, each objective needs to be comprehensively broken down into smaller chunks/principals and each task later involves critical thinking for making interpretations and judgments of the data. This is how when an employee thinks critically, he makes tradeoffs and chooses the best alternatives, and it leads to the successful achievement of KPIs. So, critical thinking is must-have for fresh graduates to become successful…”* (Employer/Recruiter K)

In light of these findings, critical thinking was also counted as an advanced-level employability skill that dictates an individual's ability to think critically and creatively, solve problems, reflect on options, and make sound decisions through proper reasoning.

### Theme 5. Social development and interaction

This theme addressed all of the specific skill sets which are needed for a graduate's social development and interaction. Employers nominated graduates' networking abilities, cultural intelligence, leadership skills, and negotiation ability as sub-elements of the skill, titled, “Positive Politics”; and the other skill identified in this theme was “team working.”

#### Positive politics

The first key skill in this theme was discovered as “Positive Politics.” The thematic analysis revealed that employers were categorized according to their opinion and views on the need for political skills in the workplace. Mostly, the discussion was loaded with numerous incidents of negative political behaviors which were notified by the employers as detrimental to the psychological and physical health of the employees and the organization. This has also been documented in the literature that politics is often practiced negatively in organizations. Therefore, to counter these negative political behaviors, the panelists emphasized the need for recruiting employees with positive political behaviors, which according to Ferris et al. (2007) further comprises attributes like “social astuteness, interpersonal influence, networking ability, and apparent sincerity.”

Most of the interviewees lodged their complaints regarding academia's failure to polish positive political behaviors and orientation in their graduates. One response in this regard was made as follows:

“*…See, political jockeying is inevitable in organizations since there is a power and resource game at the workplace. Organizations undergo continuous reorganizations in the form of downsizing, new teams and bosses, employee transfers in and transfers out. So, at the time of the interview, we calculate the person-organization fit of an employee based on his views and attitudes. In my experience, I have witnessed that too much-opinionated employees are inclined to become part of employee union groups and coalitions, bring in their bargain power over agendas, manipulate information, and are more prone to have derogatory views for the other party. We simply cannot afford employees who may tarnish the organizational image…”* (Employer/Recruiter I)

Similar to this, another eminent respondent quoted that graduates are unfamiliar with positive political skills like “good diplomacy,” a response referring it was noted as follows:

“*…In our career, we have realized that graduates have a vague idea about positive political skills which they would require in the starting phase of their careers. Like diplomacy is political skill which is required in leadership role which require decision making, evaluation of risks, providing reinforcements and resolving conflicts…”* (Employer/Recruiter G)

Furthermore, the panelists considered that graduates who are good at political skill can negotiate well and resolve conflicts. It was elaborated by a respondent as:

“*…We have witnessed that negotiation skills assure better relationships, deliver lasting and quality solutions to reach agreement and mutual satisfaction. For every negotiation style, whether it is competing, collaborating, avoiding or compromising, or accommodating, the main characteristics are credibility and influence. So even if graduates do not master negotiation, they should at least bring a caliber to influence and deal things with integrity…”* (Employer/Recruiter No. I)

Thus, negotiation was also nominated as an effective positive political skill which was considered an essential ability to reach agreements on bargainable topics by effective communication and persuasion and creating optimum value for all of the concerned parties.

Quoting a couple of other instances where positive political behaviors are needed, one respondent commented:

“*…People with the mastery of positive political skill make deals out of conflicts by understanding the informal processes of conflicts, negotiating, adapting behaviors, successfully liaising, forming working relations with competitors, and understanding of cultural norms, values, customs, and beliefs of various groups and coalitions. In addition, they master the art of publicizing one's accomplishments and complimenting others for their efforts…”* (Employer/Recruiter G)

Whereas another popular response which was agreed upon by a majority of panelists was regarding how influence, compassion, and relationship building can be practiced as a positive political attitude, especially with regard to collaboration between senior and junior employees of an organization, this comment appeared as follows:

“*…In the modern era, our youth shows up excellent digital and computer skills, therefore, at times we educate our seniors through the process of reverse mentoring. In such situations, very young employees with little experience have to act as a mentor and coach the older ones who are less Tec savvy. This situation requires different nature of political skills like influence, compassion, and relationship building…”* (Employer/Recruiter A)

Altogether, the discussion with the respondents unveiled that respondents with positive political behaviors create a positive organizational image and achieve individual and organizational benefits by using power, forming networking, social linkages, and coalitions (this coincides with the findings of Waggoner, 2020).

### Theme 6. Industrial competence

The sixth and last theme of this study uncovered how employers defined industrial competence as an essential skill of an ideal employable persona. Yet, most of the employers felt that graduates were not meeting the industrial requirements in some areas such as Work Ethics and Professionalism; and Commercial Awareness.

#### Work ethics and professionalism

Another key employability element, reported by the employers included Work ethics and Professionalism. Professionalism, in general, refers to organizational protocols, behaviors, and expectations regarding work (Williams et al., 2019), however, the panelists discussed it primarily with regard to work ethics - morally good and bad behaviors in a profession. The literature also portrays evidence for how work ethics is viewed as synonymous with professionalism (which coincides with the finding of Asio et al., 2019). Some studies have denoted it by an array of other attributes such as honesty, integrity, work ethics, and independent decision-making (Little and McMillan, 2014). Others have attributed it to basic business etiquette which includes how individuals look, dress, speak, and behave in work settings (Nair and Mukherjee, 2015). However, employers from the panel discussions limited it to the aspects of ethical and moral awareness, ethical reflection, punctuality, character, integrity, cooperation, respect, self-discipline, and professional code of conduct only. A few comments depicting these attributes are portrayed in a comment as follows:

“*…Despite possessing academic excellence, our Graduates come up with a major soft skills gap due to which they appear less professional for real-time work practices. Our industry requires a strong professional attitude, especially in the areas of punctuality, work ethics, casual work attitude, cooperation with fellow workers, and character …”* (Employer/Recruiter I)

Viewing grooming as fundamental to the work ethic practice in an organization, a respondent commented:

“*…The organizational image is dictated by the grooming, etiquettes, character, and ethics of its employees in formal and informal circumstances. Like, a sound social etiquette can be observed through the salutations used while employees address their fellow workers. Then, another ethical behavior can be the employees' concern for organizational resources, uplifting coworkers in difficult times, using power to get things done and other similar circumstances …”* (Employer/Recruiter H)

#### Commercial awareness

The employers collectively viewed commercial awareness as a defining skill to get entry into the job market, employee promotability, and career growth. Regarding sound commercial awareness, researchers contend that one has to step beyond the disciplinary realm and bring sound exposure to current issues and social market dynamics (Selvadurai et al., 2012); along with this, one needs to bring active citizenship, professional identity, and networking (Rowe and Zegwaard, 2017; Tan et al., 2022). Such as, this defines how adaptable, resourceful, and knowledgeable one can be while interacting with industrial communities (Selvadurai et al., 2012).

When asked about the importance of commercial awareness, a respondent elaborated:

“*…Commercially aware people are proactive thinkers and valuable assets for the company. They build the business by bringing in clientele through better stakeholder and customer relationships. At times they drive businesses forward through new ideas for process reengineering, spot opportunities for resources and product development, and help reduce business risks. Certainly, this adds to the efficiency, effectiveness, and competitive advantages of the businesses. This is why it's such an important skill and in-demand by almost every organization…”* (Employer/Recruiter J)

Thus, it was explored that this skill was considered to be complementary to the “Business Skills” of the individuals, which according to Nagarajan and Edwards (2014) is defined as the knowledge of work procedures and business operations, ability to make products and service promotions, and maintenance of stakeholder relationships.

However, it was also highlighted through the discussions that the reason for the lack of exposure to the market-based realities is the university-industry lack of collaboration, it was documented as follows:

“*…In Pakistan, the industry and academia have been working in SILOS and producing their yield in their own frames. This is the reason why graduates lack in exposure to the realities of the job market, emerging trends, and fast-paced changes…”* (Employer/Recruiter J)

In this regard, the employers viewed summer internships as essential since they facilitate initial exposure to real-life work experiences. Complaining about the aptitude of fresh graduates toward commercial awareness and integration, one respondent commented:

“*…As a matter of fact, University graduates lack awareness of businesses regarding real-time scenarios and the practical knowledge of organizational environments. Whereas, in the industry, we seek graduates with strong commercial awareness so that we could add on to our competitive intelligence…”* (Employer/Recruiter A)

Similarly, another comment appeared as:

“*…If a Customer Relationship Manager does not have a background knowledge of the customers' cultural origin or their cultural communication styles…Then, he/she would not be able to deploy an appropriate Customer Relationship Management Strategy. Therefore, Commercial awareness, networking and liaisons in the market is an individual's social capital which is valued by the organizations…”* (Employer/Recruiter I)

From the above-stated comments and other relevant discussions, it was deduced that by commercial awareness the respondents meant the knowledge of the business operations, market dynamics, and industry in which business competes. They also referred it to as keeping oneself updated about organizational operations, micro and macro environmental challenges, competitors, and other stakeholders.

## Conclusion

This study provides useful insight for the political discourses, reports, and papers questioning and urging the need to focus on the employability skills of Pakistani Youth so as to reap the benefits of the country's strategic position of demographic dividend. Doing so, this study presents 10 skills that are the preference of employers across the region of Bahawalpur and Rahim Yar Khan, South Punjab. Although some of these skills resonated and overlapped with the other themes or skills mentioned under other themes, the study sorted out the skills, in the simplest manner, through six themes, which emerged from conducting the thematic analysis: (1) Self-management and Organization, (2) Information handling and management, (3) Interpersonal Communication Skills, (4) Problem Solving, (5) Social Development and Interaction, and (6) Industrial Competence.

As for the final list of the 10 skill sets covered under the six themes, they included a combination of various hard and soft skills' elements, such as Emotional Intelligence, Flexibility and Adaptability, Multitasking, Computer Literacy, Digital Skills, Information Literacy and Data Analytics, Oral and Written Communication in English and Urdu, Critical Thinking, Positive Politics, Work Ethics and Professionalism, and Commercial awareness. Nonetheless, these skills were not ranked in the order of importance or preference by employers from various sectors.

## Implications

The identification of employability skills, with the help of employers, for entry-level graduate jobs, serves as an important ground for the unemployed Millennial youth (born between 1982 and 2001) of Pakistan to overcome their employability skills deficit by acquiring these skills which are in demand by the employers, particularly in the post-COVID times of “new normal.” Surely, this would not only assist their market penetration in the local and global markets but also, would be beneficial for their professional wellbeing and sustainable employment. As such, the findings of this study further lay implications for the universities across the country to devise a direct intervention for skills-specific education and training instead of the old “one size fits all” curriculum-based study.

## Limitations

The expansion of geographical scope, with a larger coverage of respondents from various industries, is suggested so as to obtain a generalized set of skills across different industries at the national level. This can also be continued conducted with a consideration of sector-specific skill sets that employers seek in the graduates since researchers such as Hinchliffe and Jolly (2011) posited that the perspective and understanding of the graduates' identity and employability varies from sector to sector. Furthermore, the scope of the study can be expanded with a specific focus on occupational skill sets; this would lead to an understanding of the professional wellbeing of employees more clearly.

## Data availability statement

The original contributions presented in the study are included in the article/supplementary material, further inquiries can be directed to the corresponding author.

## Ethics statement

The studies involving human participants were reviewed and approved by University Research Ethics Committe-The Islamia University of Bahawalpur; Approval no. 450/AS&RB. Written informed consent for participation was not required for this study in accordance with the national legislation and the institutional requirements.

## Author contributions

JI: conceptualization, supervision, and project administration. AS: writing-original draft preparation and formal analysis. WJ: supervision, review, and editing. KA: funding acquisition and arrangements of panel discussions. RR: analysis and investigation. SK: resources and investigation. All authors contributed to the article and approved the submitted version.

## Appendix 1

### Interview protocol

1. Other than the discipline-specific knowledge, are there any other criteria which you seek in the prospective employees during their selection interviews at your organization?

2. Do the fresh graduates which you recruit have adequate skills to perform in the current jobs?

3. Do you believe that soft skills are just as important as hard skills/technical skills?

4. Do you think your prospective employees are apt at self-management and organization skills?

5. In the modern era of competition, how important is it for a fresher to manage themselves well during the time of stress?

6. Do you think that for some positions it is crucial to hire a person with a good EI quotient?

7. How often do you experience that a good emotional quotient could help people overcome difficult situations?

8. Do you believe that ability to think outside of box is important in todays' dynamic and volatile business environment?

9. How do you believe “openness to experience” trait adds value to the worth of a fresh job incumbent?

10. In your view, is resilience and quick adaptation essential in the modern world when businesses transition unpredictably?

11. Do you encourage an all-rounder-multitalented employee who performs various organizational functions?

12. How important is for a job seeker to keep him/herself updated about the technologies related to a job role which one aspires for?

13. Do you feel that graduates fail to understand technological systems, and fail to correct problems in technological operations of the job, when newly hired?

14. Do you believe that organizing, maintaining, interpreting and communicating information with apt data analytical skills using computers is a key technical skill?

15. Do you face difficulty as an employer if the graduates are not well-versed at written and oral communication skill?

16. Do you think having being able to locate, understand and interpret information in company documents such as minutes, manuals, reports, graphs, and flowcharts, an important requirement of the industry?

17. What are the key attributes of a good presenter which you look for in your prospective employees?

18. In what ways do you consider critical thinking helps employees in finding solutions and making better decisions?

19. What political behaviors would you encourage or discourage at the workplace?

20. Do you find that employees getting involved in positive politics is the requirement of modern workplace?

21. Does it matter to you to hire a graduate who is commercially aware of the surrounding market and competition?

22. Do activities that may include conducting market surveys, researching the market for new references, and establishing linkages with the industry stakeholders, are a key requirement of current business survival?

23. Do you consider entrepreneurial ability as a key skill for freshers in particular?

24. To what extent do you believe that professionalism of employees adds on to the organizational character?

25. In your opinion, how important is it for a job incumbent to demonstrate integrity/honesty and choose ethical courses of action?

26. Do you think that it is a need of the time for one to be professional in working with teams with people from diverse backgrounds?

27. Can you exemplify some of the traits you particularly regard important for professionalism in the modern era?
